# Genome‐wide association analysis identifies *APOE* as a mitophagy modifier in Lewy body disease

**DOI:** 10.1002/alz.70198

**Published:** 2025-05-01

**Authors:** Xu Hou, Michael G. Heckman, Fabienne C. Fiesel, Shunsuke Koga, Alexandra I. Soto‐Beasley, Jens O. Watzlawik, Jing Zhao, Rebecca R. Valentino, Patrick W. Johnson, Launia J. White, Zachary S. Quicksall, Joseph S. Reddy, Jose Bras, Rita Guerreiro, Na Zhao, Guojun Bu, Dennis W. Dickson, Owen A. Ross, Wolfdieter Springer

**Affiliations:** ^1^ Department of Neuroscience Mayo Clinic Jacksonville Florida USA; ^2^ Division of Clinical Trials and Biostatistics Mayo Clinic Jacksonville Florida USA; ^3^ Neuroscience PhD Program Mayo Clinic Graduate School of Biomedical Sciences Jacksonville Florida USA; ^4^ Department of Quantitative Health Sciences Mayo Clinic Jacksonville Florida USA; ^5^ Department of Neurodegenerative Science Van Andel Institute Grand Rapids Michigan USA; ^6^ Division of Psychiatry and Behavioral Medicine Michigan State University College of Human Medicine Grand Rapids Michigan USA

**Keywords:** autophagy, GWAS, mitochondria, Parkin, Parkinson's disease, phosphorylated ubiquitin, PINK1, PRKN, ubiquitin, ZMIZ1

## Abstract

**INTRODUCTION:**

Phosphorylated ubiquitin (p‐S65‐Ub) is generated during PINK1‐PRKN mitophagy as a specific marker of mitochondrial damage. Despite the widespread deposition of p‐S65‐Ub in aged and diseased human brain, the genetic contribution to its accumulation remains unclear.

**METHODS:**

To identify novel mitophagy regulators, we performed a genome‐wide association study using p‐S65‐Ub level as a quantitative trait in 1012 autopsy‐confirmed Lewy body disease (LBD) samples.

**RESULTS:**

We identified a significant genome‐wide association with p‐S65‐Ub for rs429358 (*apolipoprotein E ε4* [*APOE*4]) and a suggestive association for rs6480922 (*ZMIZ1*). *APOE*4 was associated with higher p‐S65‐Ub levels and greater neuropathological burden. Functional validation in mouse and human induced pluripotent stem cell (iPSC) models confirmed *APOE*4‐mediated mitophagy alterations. Intriguingly, *ZMIZ1* rs6480922 was associated with lower p‐S65‐Ub levels, reduced neuropathological load, and increased brain weight, indicating a potential protective role.

**DISCUSSION:**

Our findings underscore the importance of mitochondrial quality control in LBD pathogenesis and nominate regulators that may contribute to disease risk or resilience.

**Highlights:**

- p‐S65‐Ub levels were used as a quantitative marker of mitochondrial damage.
- A GWAS identified two genetic variants that modify mitophagy in LBD autopsy brain.
- *APOE*4 was associated with increased p‐S65‐Ub accumulation and neuropathology.
- *APOE*4 altered mitophagy via pathology‐dependent and pathology‐independent mechanisms.
- *ZMIZ1* was linked to reduced p‐S65‐Ub and neuropathology indicative of protection.

## 1 BACKGROUND

Lewy body disease (LBD) is the neuropathological result of the abnormal accumulation of alpha‐synuclein (αSyn) in the form of Lewy bodies and Lewy neurites in the brain.^1^ Many LBD autopsy cases also exhibit varying degrees of Alzheimer's disease (AD) copathology including amyloid beta (Aβ) senile plaques (SPs) and neurofibrillary tangles (NFTs) formed by hyperphosphorylated tau.^1^ It is estimated more than six million individuals globally are affected by clinical disorders with LBD outcomes (Parkinson's disease [PD], PD with dementia [PDD], and dementia with Lewy bodies [DLB]), and there is no cure or effective therapy to slow disease progression as current treatments only relieve symptoms.^2^

To better understand disease etiology and explore new clinical biomarkers and drug targets, efforts from multiple genome‐wide association studies (GWASs) have consistently demonstrated a significant genetic contribution to the development of PD and DLB.^3^, ^4^, ^5^ These clinical conditions share overlapping common genetic risk factors, including variation in the *SNCA* and *GBA* genes, but also have distinct genetic components such as the *apolipoprotein E ε4* (*APOE*4) genotype, which is a strong risk factor for DLB but not PD, and the *MAPT* H1 haplotype, a strong risk factor for PD but not DLB.^3^, ^4^, ^5^ However, although genetic susceptibility factors for clinical Lewy body disorders have been rigorously studied, genetic modifiers of neuropathological features in LBD are not well understood. The most prominently affected biological pathways that contribute to LBD include alterations in lipid homeostasis, mitochondrial dysfunctions, and lysosomal impairments.^6^

The ubiquitin (Ub) kinase‐ligase pair PINK1‐PRKN directs a cytoprotective quality control pathway by selectively removing defective mitochondria to maintain a healthy and functional mitochondrial pool.^7^ Upon stress and in a dynamic process, PINK1‐PRKN label terminally damaged mitochondria with phosphorylated Ub (p‐S65‐Ub), which leads to their swift elimination via the autophagic–lysosomal system (mitophagy). However, it is important to recognize that the normally only transient p‐S65‐Ub signal can become more persistent due to continuing mitochondrial damage and/or an overwhelmed autophagic–lysosomal system. While complete loss of *PINK1* or *PRKN* gene function causes autosomal recessive early‐onset PD,^7^ more commonly there is an abnormal build‐up of p‐S65‐Ub labeled dysfunctional mitochondria with normal aging and with disease. In autopsy brain, p‐S65‐Ub levels were strongly associated particularly with the early stages of LBD and AD neuropathologies.^8^, ^9^, ^10^ Given that impaired mitophagy might contribute to Lewy body (LB) formation and neurodegeneration,^6^ p‐S65‐Ub level may serve not only as a marker for the PINK1‐PRKN pathway but also as a new, quantitative trait in LBD.

**FIGURE 1:**
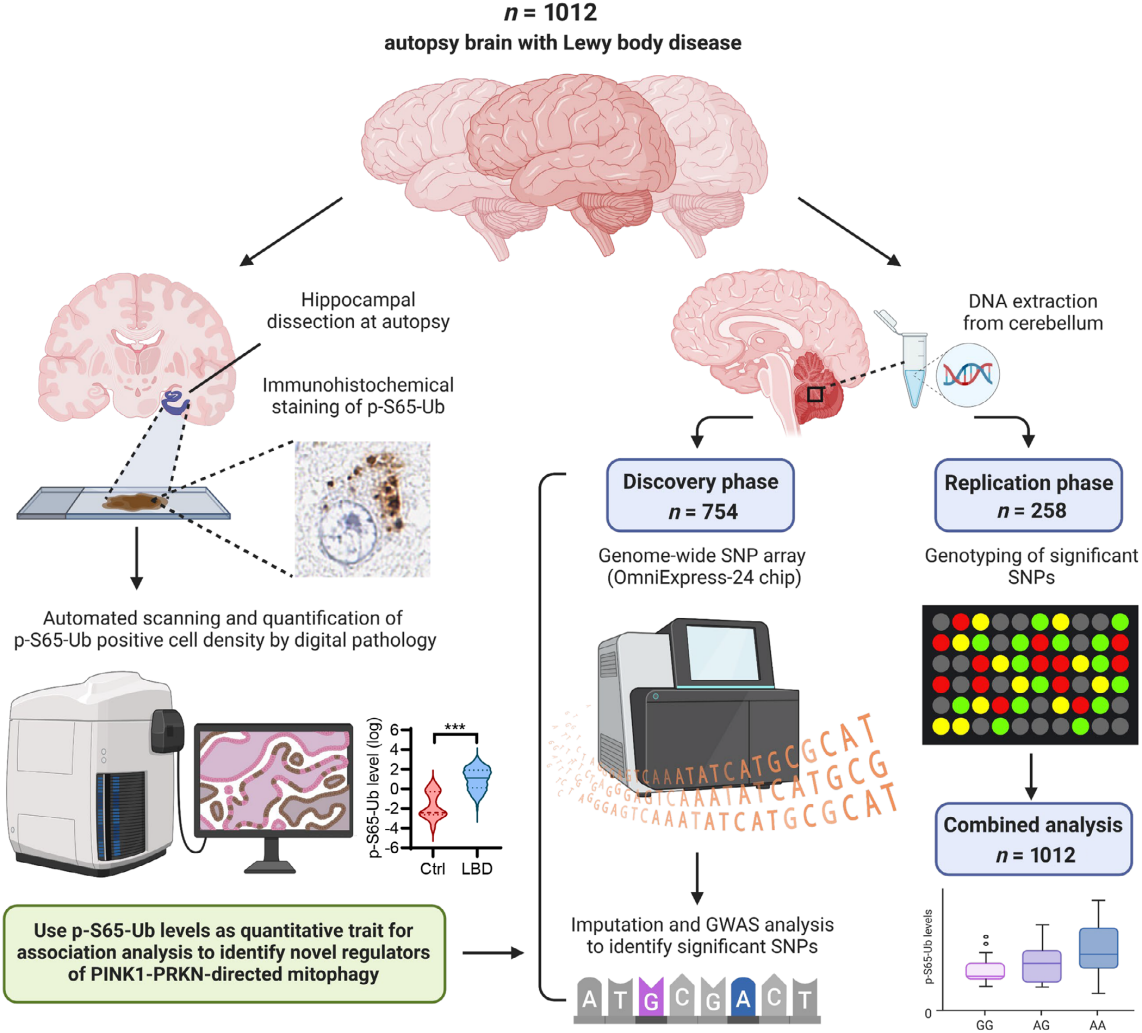
Workflow of GWAS analysis using hippocampal p‐S65‐Ub levels as quantitative trait. A total of 1021 autophagy brains with pathological confirmed LBD were obtained from the brain bank. Hippocampal region was dissected from the fixed hemibrain during autopsy and immunostained for p‐S65‐Ub by autostainer. Stained hippocampal sections were then scanned and p‐S65‐Ub‐positive cell density were quantified using unbiased digital pathology. Representative images of p‐S65‐Ub‐positive cells and levels of hippocampal p‐S65‐Ub cell density in control and LBD cases were shown. DNA was extracted from frozen cerebellar tissue from the same cohort. In the discovery phase, genome‐wide SNP array was performed in 754 LBD cases. p‐S65‐Ub levels were used a quantitative trait for genome‐wide association analysis to identify regulators of PINK1‐PRKN‐directed mitophagy. In the replication phase, significant SNPs were genotyped in the remaining 258 LBD cases. Finally, a combined analysis was performed in the entire cohort. This figure was created with BioRender.com. GWAS, genome‐wide association study; LBD, Lewy body disease; p‐S65‐Ub, phosphorylated ubiquitin.

To dissect the underlying genetic architecture linked to mitophagy alterations, herein we performed a large‐scale GWAS and replication of p‐S65‐Ub levels in 1012 pathologically confirmed autopsy LBD brains (Figure 1). We selected the hippocampus as a region central to aging and affected across a spectrum of diseases, but relatively preserved in LBD, and identified significant associations for *APOE* rs429358 (*APOE*4) during both discovery and replication and for *ZMIZ1* rs6480922, but only in a combined analysis. We then evaluated the potential neuropathological changes associated with the identified variants and functionally validated pathology‐independent *APOE*4 effects on mitophagy in mouse and in human induced pluripotent stem cell (iPSC) models. Together, our study provides important new insight into mitochondrial/lysosomal dysfunction in LBD pathogenesis and identifies potential therapeutic targets for early disease management.

## 2 MATERIALS AND METHODS

### 2.1 Sex as a biological variable

Our study included both male (*n *= 636) and female (*n *= 376) autopsy LBD cases. All analyses involving human brain samples were adjusted for sex. Our study examined male and female animals, and similar findings are reported for both sexes.

**RESEARCH IN CONTEXT**

1. **Systematic review**: Genetic studies in Lewy body disease (LBD) highlight the contribution of mitochondrial and lysosomal dysfunction to the pathogenesis. p‐S65‐Ub, a specific marker of mitochondrial damage during PINK1‐PRKN mitophagy, accumulates in aged and diseased human brain. However, the genetic factors underlying p‐S65‐Ub deposition remain unclear.
2. **Interpretation**: Capitalizing on p‐S65‐Ub as a selective mitophagy label and quantitative trait, we identified *apolipoprotein E ε4* (*APOE*4) and *ZMIZ1* rs6480922 as novel modifiers in a LBD autopsy brain GWAS. As mitochondrial and lysosomal deficits are early disease features, our study highlights the importance of mitochondrial quality control in disease pathogenesis and identifies potential therapeutic targets for early disease management.
3. **Future directions**: Follow‐up studies should explore mechanisms underlying *APOE*4‐mediated mitophagy alterations and validate *ZMIZ1* rs6480922 protective role against mitochondrial damage and neuropathology. Future research should assess genetic mitophagy modifiers across diverse ethnic groups, brain regions, and disease cohorts to reveal shared and distinct pathogenic pathways.

### 2.2 Study design and subjects

A total of 1012 LBD autopsy brains that were obtained from the Mayo Clinic Florida brain bank between 1998 and 2021 were included and divided into discovery series (*n *= 754) and independent replication series (*n *= 258). All cases were unrelated and self‐reported non‐Hispanic Caucasians. All brains were examined in a systematic and standardized manner by a single neuropathologist (DWD). Available demographic and neuropathological information included age at death, sex, brain weight, LBD subtype (brainstem, transitional, or diffuse), Braak NFT stage (0 to VI), and Thal amyloid phase (0 to 5), as well as αSyn burden (by immunohistochemistry) and densities of SPs and NFTs (by thioflavin S) in the hippocampus (Tables S1 and S2).

### 2.3 Genome‐wide association and replication

Frozen cerebellum brain tissue was collected from all cases and genomic DNA was extracted using Autogen Flex Star methods. LBD cases from discovery series were genotyped in the Illumina OmniExpress‐24 genotyping array (Illumina). Subject‐level exclusion criteria based on GWAS data included a call rate <98%, significant relatedness (PI_HAT > 0.25), and non‐Caucasian ancestry as determined by ADMIXTURE.^11^ Variants were excluded if they had a call rate <98%, a Hardy–Weinberg equilibrium (HWE) *p *< 1 × 10^−5^, or a minor allele frequency (MAF) <1%. Genome‐wide imputation was performed with the TOPMed Imputation Server, where a genotype call (i.e., 0, 1, or 2 copies of the minor allele) was made for each variant based on imputed genotype probabilities.^12^ Following imputation, the aforementioned variant‐level exclusion criteria were utilized, with the addition of excluding variants with an imputation *r* ^2^ < 0.8; this resulted in a total of 8,696,291 variants available for analysis. In the replication series, all samples were genotyped using a custom‐designed MassARRAY System iPlex assay and 2 custom Taqman SNP genotyping assays (rs429358 and rs76354500). All genotype call rates were >99%, and there was no evidence of a departure from HWE (all *p *≥ 0.10).

### 2.4 Statistical analysis of GWAS data

For the discovery and the replication series, single‐variant associations with p‐S65‐Ub level were examined using linear regression models that were adjusted for age at death, sex, hippocampal region, and the top five principal components (PCs) of genetic data (the top five PCs were not adjusted for in the replication series). p‐S65‐Ub level was assessed on the natural logarithm scale owing to its skewed distribution. Variants were examined under an additive model (i.e., effect of each additional minor allele). Regression coefficients (denoted by *β*) and 95% confidence intervals (CIs) were estimated and are interpreted as the change in p‐S65‐Ub level (on the natural logarithm scale) per each additional minor allele of the given variant. For variants that were included in both the discovery series and the replication series, the results of association analyses were combined to obtain regression coefficients, 95% CIs, and *p* values using a random‐effects meta‐analysis with inverse‐variance weighting. The results of this meta‐analysis combining the two series were considered to be the primary analysis, given that joint analysis of GWASs with a discovery/replication approach has been shown to be more efficient than focusing on the replication series alone (see Figure 1 for an overview of the workflow).^13^

In the discovery series and in the combined analysis, *p *< 5 × 10^−8^ was considered as genome‐wide significant, while *p *< 1 × 10^−6^ was considered as displaying suggestive evidence of an association. *P* values < 0.05 were considered statistically significant in the replication series. All statistical tests were two‐sided. Our analysis code is available at https://github.com/ORossLab/PhosphoUb‐GWAS, and statistical analyses were performed using PLINK version 1.9 and R Statistical Software (version 4.1.2).

### 2.5 Immunohistochemistry and image analysis

Immunohistochemical staining of paraffin‐embedded *post mortem* brain tissue containing anterior or posterior hippocampus was performed as previously described.^8^ The tissue blocks were collected and prepared in a very uniform way, ensuring a consistent and comparable hippocampal region in all samples. The paraffin‐embedded brain tissue was cut into 5‐µm sections and allowed to dry overnight at 60°C. Slides were deparaffinized and rehydrated, followed by antigen retrieval in steaming deionized water. After blocking with 0.03% hydrogen peroxide and 5% normal goat serum (Invitrogen, 16210072), sections were incubated with primary antibodies against p‐S65‐Ub (in‐house, 1:650), followed by rabbit‐labeled polymer HRP (Agilent, K4011) at room temperature. Peroxidase labeling was visualized with the DAB chromogen before sections were counterstained and coverslipped. After staining, all sections were scanned with an Aperio AT2 digital pathology scanner (Leica Biosystems) and then traced and quantified using optimized Aperio algorithms to count the positive cell number followed by manual quality control.

### 2.6 *APOE*‐targeted replacement mice and Meso Scale Discovery electrochemiluminescence assays

*APOE*‐targeted replacement (*APOE*‐TR) mice in which murine *Apoe* gene locus is replaced with human *APOE* gene (either *ε* *3* or *ε* *4* allele) were purchased from Taconic and used here.^14^ Age‐ and sex‐matched *APOE*‐TR mice were anesthetized at 3 or 22 months of age (*n *= 7 or 8 mice/genotype/age group). Brains were quickly removed after transcardial perfusion with PBS (pH 7.4), divided along the sagittal plane, and then snap‐frozen in liquid nitrogen. For protein extraction, hemibrain from *APOE*‐TR mice were homogenized in RIPA buffer (50 mM Tris, pH 8.0, 150 mM NaCl, 0.1% SDS, 0.5% deoxycholate, 1% NP‐40). After 30 min incubation on ice, samples were centrifuged for 15 min at 14,000 rpm at 4°C, and the supernatant was collected as total brain lysate. Protein content was determined by bicinchoninic acid (BCA) assay (Thermo Fisher Scientific, 23225).

p‐S65‐Ub levels were measured on a Meso Scale Discovery (MSD) system as previously described.^15^ Plates of 96 wells were coated with the p‐S65‐Ub antibody (Cell Signaling Technology, 62802, 1:100) in carbonate coating buffer (200 mM Na2CO3, pH9.6), incubated overnight at 4°C, and washed before use. Following 1 h blocking with 1% BSA in TBST, 30 µg of brain lysates were diluted in 1% BSA in TBST to 30 µL and loaded into each well in duplicate. Samples were incubated for 2 h in the plate at room temperature while shaking. The plates were then washed again before incubating with the total Ub detection antibody (Thermo Fisher Scientific, 14‐6078‐82, 1:100) for 2 h at room temperature. After three washes, SULFO‐TAG detection antibody (Meso Scale Discovery, R32AC, 1:500) was incubated in the plate for 1 h at room temperature. After the final wash, MSD Gold read buffer (Meso Scale Discovery, R92TG) was added to each well, followed by plate reading on the MESO QuickPlex SQ 120 (Meso Scale Diagnostics).

### 2.7 Differentiation of human iPSCs into astrocytes and Western blot

Human iPSCs from normal individuals with a homozygous *APOE ε3/ε3* or *ε4/ε4* genotype were generated as previously described.^16^ To generate neural progenitor cells (NPCs), the iPSC clumps were cultured in neural induction medium (Stemcell Technologies) in suspension for 5 to 7 days to initiate neurosphere formation. Next, neurospheres were seeded onto Matrigel‐coated dishes and cultured in neural induction medium for another 5 to 7 days to induce neural rosette formation. Neural rosettes were isolated as a single‐cell suspension and replated onto Matrigel‐coated dishes in neural induction medium. The medium was then replaced with neural progenitor cell medium (StemCell Technologies) and cultured for 10 to 14 more days to induce NPC differentiation. For astrocyte differentiation, NPCs were cultured on Poly‐D‐Lysine‐coated plates in astrocyte differentiation medium composed of astrocyte medium (ScienCell) with CNTF (10 ng/mL), BMP4 (10 ng/mL), and Heregulin‐β (10 ng/mL) (all from Stemcell Technologies). At day 30, the NPC‐derived astrocytes were treated with 10 µM carbonyl cyanide m‐chlorophenyl hydrazone (CCCP; Sigma Aldrich, C2759) for 0 or 8 h and then lysed in RIPA buffer containing protease inhibitor cocktail and phosphatase inhibitors (Sigma‐Aldrich, 11697498001 and 04906837001). Cell lysates were incubated on ice for 30 min and centrifuged in cold for 15 min at 14,000 rpm to remove the cell debris. Supernatants were collected and protein concentrations were determined by BCA assay (Thermo Fisher Scientific, 23225).

Cell lysates containing 20 µg of protein were diluted in Laemmli buffer (62.5 mM Tris, pH 6.8, 1.5% SDS, 8.33% glycerol, 1.5% β‐Mercaptoethanol, 0.005% bromophenol blue) and boiled at 95°C for 5 min. The denatured samples were then run on Tris‐Glycine gels (Invitrogen, EC60485BOX). After transferring protein onto PVDF membranes (Millipore Sigma, IPVH00010), the membranes were blocked in 5% skim milk (Genesee, 20‐241) and incubated with primary antibodies against p‐S65‐Ub (in‐house, 1:10,000), PINK1 (Cell Signaling Technology, 6946; 1:1,000), and GAPDH (Meridian Life science, H86504 M; 1:150,000) overnight at 4°C, followed by secondary HRP‐conjugated antibodies (Jackson ImmunoResearch, 711‐035‐152 and 715‐035‐150, 1:10,000) for 1 h at room temperature. Protein bands were visualized using Immobilon Western Chemiluminescent HRP Substrate (Millipore Sigma, WBKLS0500) and Blue Devil Lite X‐ray films (Genesee Scientific, 30‐810L).

### 2.8 Other statistical analysis

Associations of *APOE* rs429358 (*APOE*4) and *ZMIZ1* rs6480922 with brain weight, αSyn burden, and densities of SPs and NFTs were examined using linear regression models with adjustment for age at death and sex. αSyn burden was evaluated on the natural logarithm scale while SP and NFT density was examined on the cube root scale owing to the skewed distributions of these measures. *β* coefficients and 95% CIs were estimated and are interpreted as previously described. For biochemical measures in animals and cells, unpaired *t*‐tests were used for comparison between *APOE ε3* (*APOE*3) and *APOE*4 groups, and *p *< 0.05 was considered statistically significant. All statistical tests were two‐sided. Statistical analyses were performed using R Statistical Software (version 4.1.2) and GraphPad Prism (GraphPad Software; version 9).

### 2.9 Study approval

All brain samples were from autopsies performed after approval by the legal next of kin. Research on deidentified *post mortem* brain tissue is considered exempt from human subject regulations by the Mayo Clinic Institutional Review Board (IRB). All animal procedures were approved by the Mayo Clinic Institutional Animal Care and Use Committee (IACUC) in accordance with the National Institutes of Health. Human iPSCs were collected and generated under approved IRB protocols with consent for research purpose.

### Data availability

The datasets used and/or analyzed during the current study are available at the Synapse portal or dbGAP repository. The summary statistics can be accessed through the GWAS catalogue. The analysis code is publicly available at https://github.com/ORossLab/PhosphoUb‐GWAS.

## 3 RESULTS

### 3.1 GWAS identified variants associated with hippocampal p‐S65‐Ub levels in LBD brains

**FIGURE 2:**
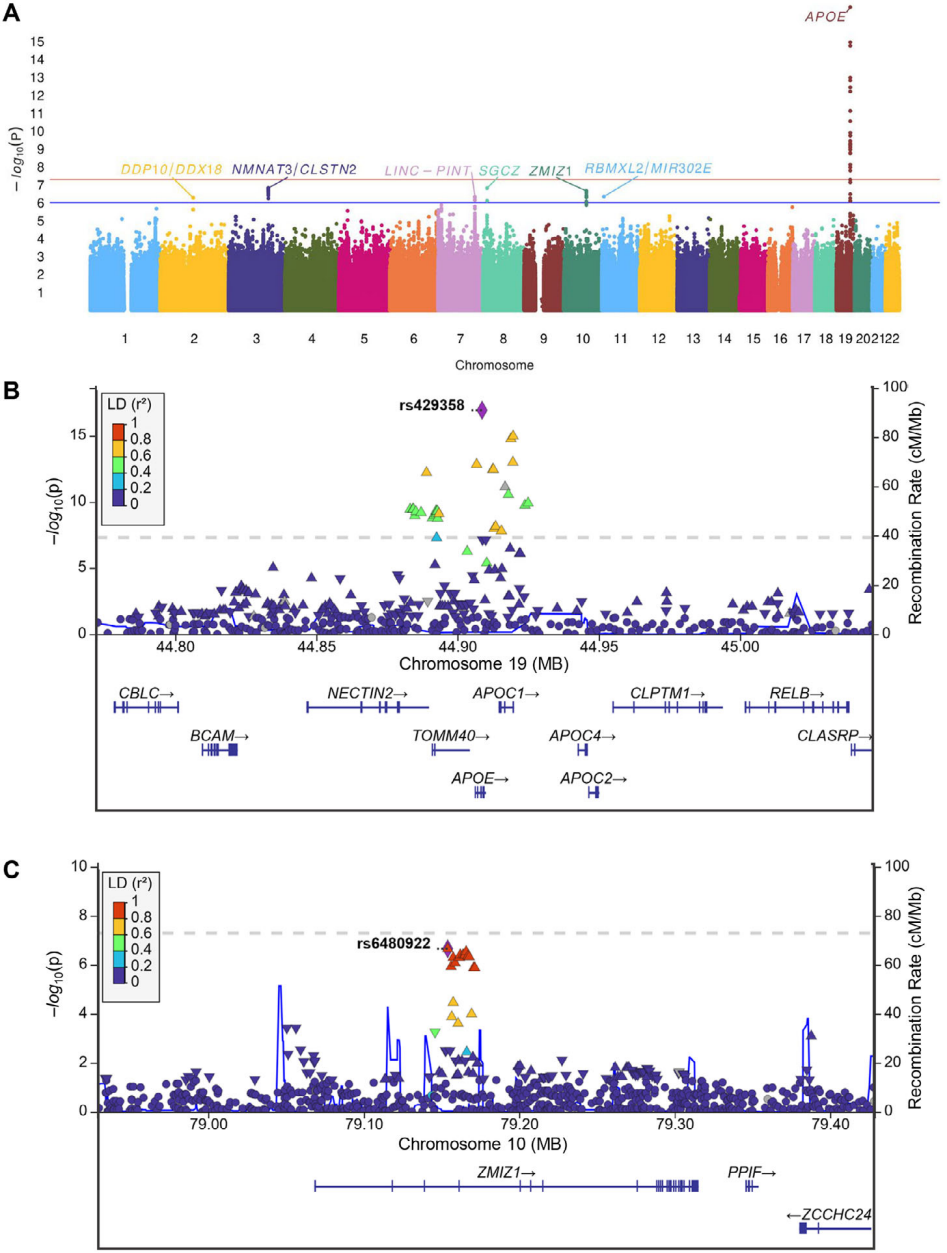
GWAS analysis identified two distinct mitophagy modifiers in LBD autopsy brain. (A) Manhattan plot showing genome‐wide *p* values of association with p‐S65‐Ub levels. *Y*‐axis: −log10 *p* values of 8,696,291 SNPs; *x*‐axis: their chromosomal positions; red solid line: *p* value threshold used to define genome‐wide significant association (*p *< 5 × 10^−8^); blue solid line: *p* value threshold used to define “suggestive” association (*p *< 1 × 10^−6^). (B and C) Locus Zoom plot showing association of variants at *APOE* (B) and *ZMIZ1* (C) loci with p‐S65‐Ub levels. The most significant variants (rs429358 and rs6480922) are indicated in purple. The association *p* value is shown on the *y*‐axis and linear position on the chromosome on the *x*‐axis. Each point on plot represents one variant; colors of points indicate linkage disequilibrium (*r* ^2^) value with index variant (rs429358 or rs6480922). Points that are missing linkage disequilibrium information are shown in gray. Our analysis code is available at https://github.com/ORossLab/PhosphoUb‐GWAS. GWAS, genome‐wide association study; LBD, Lewy body disease; p‐S65‐Ub, phosphorylated ubiquitin.

This study included a total of 1012 pathological confirmed LBD autopsy brains in the discovery (*n *= 754) and replication series (*n *= 258) with demographic information and detailed neuropathological characterization available (Tables S1 and S2). Using an established, mostly automated workflow, hippocampal sections of the LBD cases were immunostained for the mitophagy marker p‐S65‐Ub and quantified with unbiased algorithms to serve as a quantitative trait for mitochondrial damage and mitophagy alteration. In our GWAS analysis of p‐S65‐Ub levels involving 8,696,291 variants and where all regression models were adjusted for age at death, sex, hippocampal region, and the top five PCs, we observed a λ value equal to 1.020, indicating good control of population stratification. A Manhattan plot is displayed in Figure 2A.

We identified one genome‐wide significant association with p‐S65‐Ub level, *APOE* rs429358 (*β*: 0.50, 95% CI: 0.39 to 0.62; *p *= 1.18 × 10^−17^), and an additional six suggestive associations (Table 1). These seven genome‐wide significant or suggestive variants were then assessed in the replication series, where there was again a strong association between *APOE* rs429358 and p‐S65‐Ub level (*β*: 0.52, 95% CI: 0.32 to 0.72; *p *= 9.92 × 10^−7^; Table 1). Additionally, though not quite significant at *p *< 0.05, there was some evidence of an independent replication for *ZMIZ1* rs6480922 (*β*: −0.23, 95% CI: −0.48 to 0.01; *p *= 0.065; Table 1). Genotype, allele counts, and frequencies of variants identified and assessed in the discovery and replication series are shown in Supplemental Table S3a,b, respectively.

**TABLE 1 alz70198-tbl-0001:** Genome‐wide or suggestive associations with p‐S65‐Ub level.

					Discovery series (*n *= 754)			Replication series (*n *= 258)			Combined analysis (*n *= 1012)	
Chr.	Position	Variant	Gene or closest gene^a^	MA	MAF	β (95% CI)	*P*	MAF	*β* (95% CI)	*p*	*β* (95% CI)	*p*
Genome‐wide significant associations in discovery series (*p *< 5 × 10^−8^)												
19	44908684	rs429358	*APOE*	C	30.0%	0.50 (0.39, 0.62)	1.18 × 10^−17^	29.1%	0.52 (0.32, 0.72)	9.92 × 10^−7^	0.50 (0.41, 0.60)	8.67 × 10^−25^
Suggestive associations in discovery series (*p *< 1 × 10^−6^)												
2	116947120	rs6712544	*DPP10* and *DDX18* ^a^	A	4.8%	−0.64 (−0.89, −0.39)	5.33 × 10^−7^	5.8%	−0.14 (−0.57, 0.29)	0.52	−0.42 (−0.91, 0.06)	0.089
3	139926692	rs10935361	*NMNAT3* and *CLSTN2* ^a^	T	30.7%	−0.32 (−0.43, −0.20)	1.52 × 10^−7^	31.0%	−0.05 (−0.27, 0.17)	0.68	−0.20 (−0.46, 0.06)	0.13
7	130884634	rs157916	*LINC‐PINT*	A	47.9%	0.28 (0.17, 0.39)	4.69 × 10^−7^	47.1%	−0.05 (−0.24, 0.14)	0.60	0.12 (−0.20, 0.45)	0.45
8	14866463	rs76354500	*SGCZ*	C	3.8%	−0.73 (−1.00, −0.46)	1.54 × 10^−7^	4.1%	−0.28 (−0.77, 0.20)	0.26	−0.55 (−0.98, −0.12)	0.012
10	79154025	rs6480922	*ZMIZ1*	T	20.4%	−0.36 (−0.49, −0.22)	2.21 × 10^−7^	20.5%	−0.23 (−0.48, 0.01)	0.065	−0.33 (−0.45, −0.22)	1.42 × 10^−8^
11	7194782	rs11041236	*RBMXL2* and *MIR302E* ^a^	T	1.2%	−1.28 (−1.78, −0.79)	4.51 × 10^−7^	1.9%	0.52 (−0.20, 1.25)	0.15	−0.40 (−2.16, 1.36)	0.66

*Notes*: For the discovery series and the replication series, *β* values, 95% CIs, and *p* values result from linear regression models that were adjusted for age at death, sex, hippocampal region, and the top five principal components of genetic data (the top five principal components were adjusted for only in the discovery series). *β* values are interpreted as the change in mean p‐S65‐Ub level (on the natural logarithm scale) corresponding to each additional minor allele for the given variant. For the combined analysis, *β* values, 95% CIs, and *p* values result from a random effects meta‐analysis.

Abbreviations: Chr, chromosome; MA, minor allele; MAF, minor allele frequency; *β*, regression coefficient; CI, confidence interval.

^a^ The closest genes to the identified variant.

### 3.2 Combined analysis of discovery‐replication series reveals two genome‐wide significant hits

**FIGURE 3 alz70198-fig-0003:**
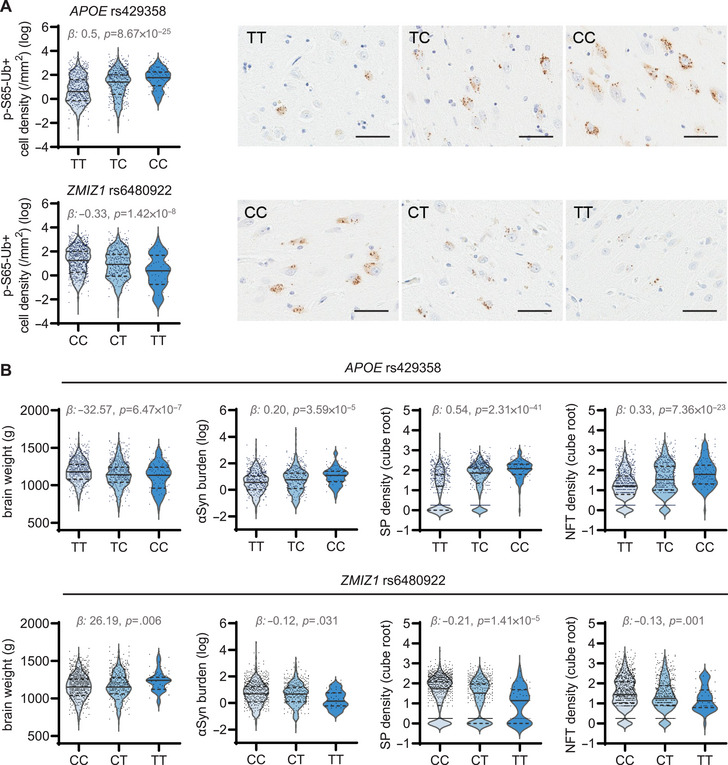
Significant association of *APOE* and *ZMIZ1* variants with p‐S65‐Ub levels, brain weight, and neuropathological measures in LBD autopsy brain. (A) Violin plot of pS65‐Ub level (natural logarithm scale) according to number of minor alleles of *APOE* rs429358 and *ZMIZ1* rs6480922 in combined (discovery and replication) series, together with the corresponding representative images of p‐S65‐Ub staining in hippocampus in LBD cases. Scale bar: 50 µm. (B) Violin plot of brain weight, αSyn burden (by NACP staining) (natural logarithm scale) and densities of SPs and NFTs (count per microscope field) (cube root scale) according to number of minor alleles of *APOE* rs429358 and *ZMIZ1* rs6480922 in the combined (discovery and replication) series. The associations were examined using linear regression models with adjustment for age at death and sex. αSyn, alpha‐synuclein; *β*, regression coefficient beta; LBD, Lewy body disease; NFT, neurofibrillary tangle; p‐S65‐Ub, phosphorylated ubiquitin; SP, senile plaque.

In addition to the GWAS and replication, we conducted a combined analysis of both cohorts as it has been shown to be more efficient than focusing on separate discovery‐replication phases.^13^ Using this approach, genome‐wide significant associations with p‐S65‐Ub level were observed for both *APOE* rs429358 (*β*: 0.50, 95% CI: 0.41 to 0.60; *p *= 8.67 × 10^−25^; Figures 2, 3 and Table 1) and *ZMIZ1* rs6480922 (*β*: −0.33, 95% CI: −0.45 to −0.22; *p *= 1.42 × 10^−8^; Figure 2, 3 and Table 1). The results of association analysis are shown separately for males and females in Tables S4 and S5.

Additionally, rather than assessing *APOE* as the number of *ε4* alleles as determined by rs429358, we incorporated information regarding *APOE* rs7412 to evaluate the association between full *APOE* genotype (i.e., *ε2/ε3*, *ε2/ε4*, *ε3/ε3*, *ε3/ε4*, *ε4/ε4*; there were no *ε2/ε2* genotypes in our study) and p‐S65‐Ub level. As shown in Table S6 and Figure S1, in combined analysis of the entire discovery/replication series, when compared to *APOE* *ε3/ε3*, p‐S65‐Ub level was modestly lower for *ε2/ε3* (*β*: −0.25, 95% CI: −0.59 to 0.09; *p *= 0.16) and *ε2/ε4* (*β*: −0.38, 95% CI: −0.74 to −0.02; *p *= 0.037) genotypes, but noticeably higher for *ε3/ε4* (*β*: 0.54, 95% CI: 0.39 to 0.68; *p *= 1.59 × 10^−13^) and especially *ε4/ε4* (*β*: 1.00, 95% CI: 0.77 to 1.22; *p *= 5.35 × 10^−18^) genotypes.

We next investigated the potential dependence between the two genome‐wide significant hits in the combined series. In linear regression analysis adjusting for both variants (as well as age at death, sex, and series), relatively consistent associations with p‐S65‐Ub level were noted for *APOE* rs429358 (*β*: 0.49, 95% CI: 0.39 to 0.59; *p *= 5.97 × 10^−22^) and *ZMIZ1* rs6480922 (*β*: −0.28, 95% CI: −0.40 to −0.16; *p *= 2.25 × 10^−6^). Interestingly, the association between p‐S65‐Ub and *ZMIZ1* rs6480922 was stronger for LBD cases without an *APOE*4 allele (*β*: −0.38, 95% CI: −0.54 to −0.22; *p *= 2.87 × 10^−6^) compared to cases who did carry *APOE*4 (*β*: −0.19, 95% CI: −0.36 to −0.02; *p *= 0.029). Of note, information regarding *post* *mortem* interval (PMI) was available for only 412 of our 1012 LBD cases, and therefore PMI was not adjusted for in any regression analyses. However, as PMI was only very weakly correlated with p‐S65‐Ub level (Spearman's *r*: 0.067), PMI would not have noticeably affected the results of any of our association analysis.[Table alz70198-tbl-0001]

### 3.3 Variants in *APOE* and *ZMIZ1* associate with changes in brain weight and neuropathological burden

Next, we aimed to examine the mechanism by which the identified *APOE* and *ZMIZ1* variants might influence hippocampal p‐S65‐Ub levels. In this context, it is noteworthy to clarify that the *APOE*4 genotype was associated with greater p‐S65‐Ub levels, while the *ZMIZ1* variant was associated with lesser p‐S65‐Ub levels (Figure 3A). Both effects were gene‐dosage dependent. From earlier studies we know that the p‐S65‐Ub level strongly correlates with the neuropathological burden in LBD and in AD.^8^, ^9^, ^10^ We thus collected additional quantitative neuropathological measures for all cases (Table S2) and studied their respective associations with the identified variants in the combined series after adjustment for age at death and sex (Table S7 and Figure 3B).

It is well established that *APOE*4 increases risk for AD and DLB and is known to exacerbate neuropathological burden but may also impact cognition independently.^17^, ^18^ Indeed, brain weight was negatively associated with increased *APOE*4 allele count (*p *= 6.47 × 10^−7^). In line, *APOE*4 was also associated with an increase of each neuropathological load as measured by αSyn burden (NACP immunostaining) (*p *= 3.59 × 10^−5^) and densities of SPs and NFTs (*p *= 2.31 × 10^−41^ and *p *= 7.36 × 10^−23^) in the hippocampus. In contrast, the *ZMIZ1* variant was significantly associated with increased brain weight (*p *= 0.0006) as well as reduced neuropathological burden of αSyn (*p *= 0.031), SP (*p *= 1.41 × 10^−5^), and NFT (*p *= 0.001).

### 3.4 *APOE*4‐associated p‐S65‐Ub increase in mouse model and iPSC‐derived astrocytes independent from neuropathology

**FIGURE 4 alz70198-fig-0004:**
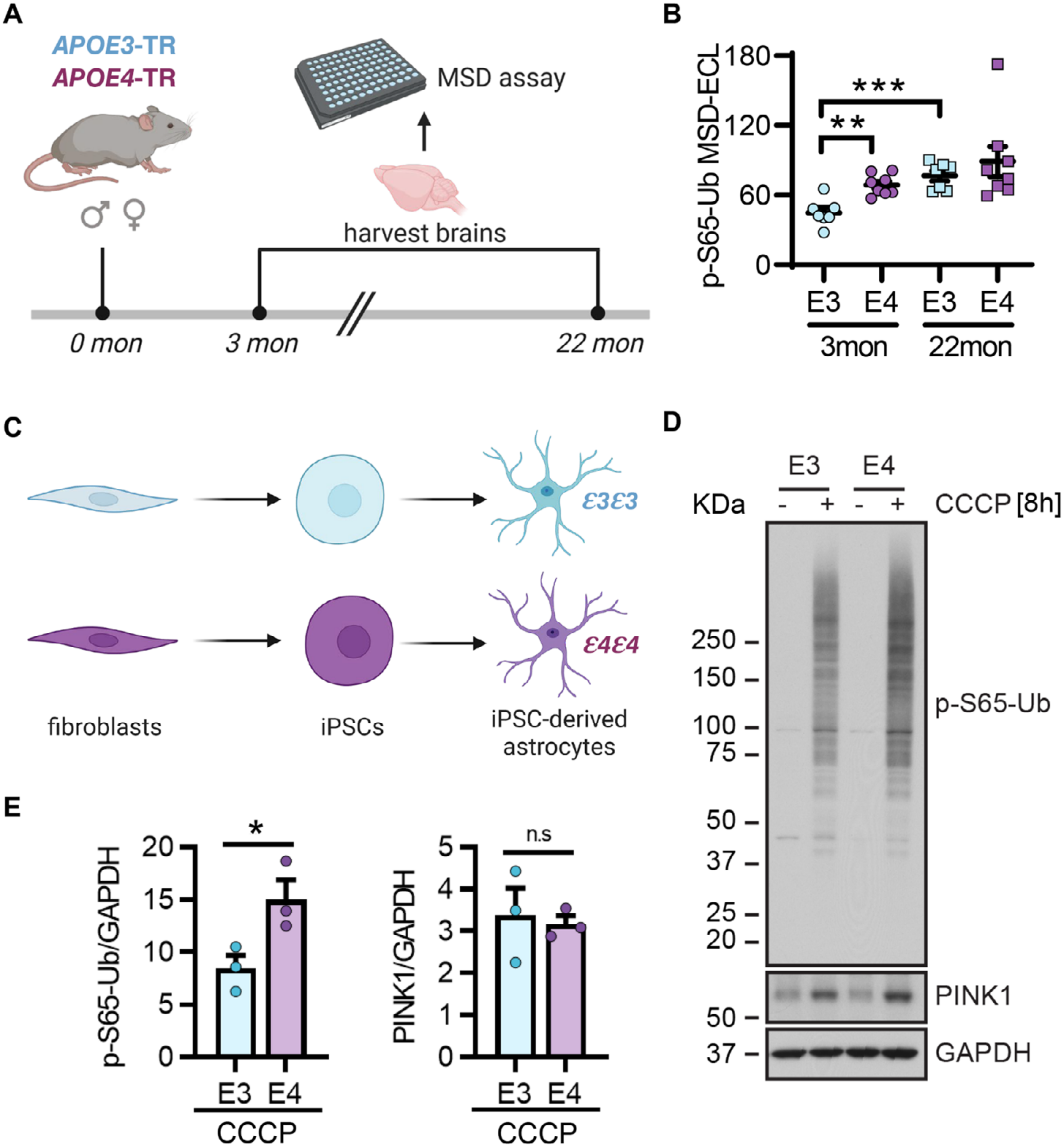
Functional validation of *APOE*4 effect on mitophagy changes in mouse and iPSC models. (A) Brain lysates from 3‐ and 22‐month‐old *APOE*‐TR mice are used to measure p‐S65‐Ub levels. (B) MSD shows significantly increased p‐S65‐Ub levels in brain lysates from 3‐months‐old *APOE*4 mice compared to age‐matched *APOE*3 mice. An age‐dependent increase of p‐S65‐Ub levels is also observed in *APOE*3 mice. *n *= 7 to 8 mice/genotype/age group. (C) Dermal fibroblasts collected from individuals carrying homozygous *APOE*3 or *APOE*4 are converted into iPSCs, which are then differentiated into astrocytes. (D) Representative Western blot images of human iPSC‐derived astrocytes. (E) Western blot results showed that human iPSC‐derived homozygous *APOE*4 astrocytes have significantly increased p‐S65‐Ub levels but similar PINK1 levels compared to homozygous *APOE*3 astrocytes upon mitochondrial depolarization (8 h CCCP treatment). *n *= 3 independent differentiation experiments. Unpaired *t*‐tests were used for comparison between *APOE*3 and *APOE*4 groups, and *p *< 0.05 was considered statistically significant. Panels (A) and (C) were created with BioRender.com. *APOE*‐TR, *APOE*‐targeted replacement; CCCP, carbonyl cyanide m‐chlorophenyl hydrazone; iPSC, induced pluripotent stem cell; p‐S65‐Ub, phosphorylated ubiquitin.

To test potential neuropathology‐independent *APOE*4 effects on mitophagy, we assessed p‐S65‐Ub levels in experimental animals and cells. Given the overall much lower levels of p‐S65‐Ub in these models compared to autopsy brain,^19^ we used a previously established sensitive MSD electrochemiluminescence assay.^15^ First we analyzed the brains of *APOE*‐TR mice, in which the murine *Apoe* gene locus is replaced with human *APOE*3 or *APOE*4 gene.^14^ We detected significantly elevated p‐S65‐Ub levels in brain lysates of *APOE*4 mice at younger age (3 months) compared to age‐ and sex‐matched *APOE*3 mice (Figure 4A,B). Additionally, p‐S65‐Ub levels were increased in aged *APOE*3 mice relative to their younger counterparts but no further escalation in aged *APOE*4 mice in comparison to both young *APOE*4 and aged *APOE*3 mice.

As *APOE* is mainly expressed in astrocytes within the central nervous system (CNS), we next examined p‐S65‐Ub levels at baseline and upon mitochondrial stress in human iPSC‐derived astrocytes from *APOE*3 or *APOE*4 homozygous individuals (Figure 4C). Treatment with the mitochondrial stressor CCCP induced significantly higher p‐S65‐Ub levels in *APOE*4 astrocytes compared to *APOE*3, while levels of Ub kinase PINK1 were similar between two genotypes (Figure 4D,E). Altogether, we were able to confirm *APOE*4‐associated p‐S65‐Ub increase in mouse brain and human iPSC models that was independent of disease‐related pathology.

## 4 DISCUSSION

Our GWAS[Fig alz70198-fig-0003] of[Fig alz70198-fig-0004] neuropathologically defined LBD cases uncovered common genetic variants that modify levels of p‐S65‐Ub, the mitophagy tag. p‐S65‐Ub, which serves as a selective and sensitive marker of mitochondrial damage and altered mitophagy flux, was used as quantitative endophenotype. We identified and replicated a strong genome‐wide significant association with p‐S65‐Ub on chromosome 19 for rs429358 (*APOE*4). A combined analysis identified another significant association on chromosome 10, rs6480922, in the *ZMIZ1* gene, which was initially only suggestive at the discovery stage. Both variants are associated with p‐S65‐Ub levels in an allele dose‐dependent manner, but in different directions: that is, *APOE* rs429358 is associated with higher‐than‐normal while *ZMIZ1* rs6480922 is associated with lower‐than‐normal levels of p‐S65‐Ub. These opposite effects were also reflected in differential associations of both variants with brain weight as well as hippocampal burden of αSyn, Aβ, and phosphorylated tau (p‐tau) pathologies. This may suggest that p‐S65‐Ub has greater sensitivity than each neuropathological parameter alone, which could allow for not only the identification of genetic modifiers of LBD but perhaps also resilience to neuropathology.

The *APOE*4 allele is the strongest common genetic risk factor for both AD and DLB.^3^, ^20^ A role for *APOE*4 in driving Aβ and p‐tau deposition is well established,^17^ and it has been suggested that *APOE*4 is also associated with increased severity and spread of Lewy bodies, independently of AD pathology.^21^, ^22^, ^23^ p‐S65‐Ub level have been shown to increase in human brain with age and independently with αSyn and early tau pathology.^8^, ^9^, ^10^ As such, it is conceivable that at least some of the *APOE*4‐dependent increase in p‐S65‐Ub could stem from an exacerbated neuropathology. However, due to the very strong association of *APOE*4 with both p‐S65‐Ub and the different neuropathologies in LBD, we cannot simply adjust for such neuropathological measures in our statistical models in order to assess whether *APOE*4 is associated with p‐S65‐Ub levels independently of the neuropathology. Therefore, we undertook an experimental model approach to better elucidate a possible independent association between *APOE*4 and p‐S65‐Ub.

The apoE protein, a major lipid carrier predominantly produced in astrocytes in the brain, broadly impacts lipid homeostasis and thereby affect functions of both mitochondria and lysosomes.^24^, ^25^, ^26^ As such it is plausible that p‐S65‐Ub levels are increased through *APOE*4‐dependent changes in lipid metabolism, which could lead to increased mitochondrial dysfunction or impaired lysosomal clearance of damaged mitochondria. To test such biological effects, we utilized *APOE*‐TR mice that express human *APOE*3 or *APOE*4 and display the characteristic lipid dysregulation, but no disease‐related pathology.^14^ p‐S65‐Ub levels were indeed significantly higher in the brains of young *APOE*4 mice (3 months) compared to age‐matched *APOE*3 mice reaching levels typically seen only at older ages (22 months). This is consistent with our prior work that demonstrated an age‐dependent increase of p‐S65‐Ub in human^8^, ^27^ and recently also in mouse brain.^19^, ^28^

Additional analyses of iPSC‐derived astrocytes treated with the mitochondrial depolarizer CCCP, which maximally activates PINK1‐PRKN signaling, further confirmed higher p‐S65‐Ub levels in *APOE*4 compared to *APOE*3 astrocytes. Of note, the kinase PINK1 that generates the p‐S65‐Ub signal was similarly stabilized following CCCP treatment, potentially pointing toward a less effective clearance of p‐S65‐Ub‐labeled mitochondria in *APOE*4 astrocytes. However, additional investigations of the underlying mechanism(s) in astrocytes and other CNS cell types including neurons will be important to discriminate between enhanced induction of mitophagy and a blockage of autophagic–lysosomal flux. In summary, we found significantly elevated p‐S65‐Ub levels in complementary experimental models in the absence of αSyn, Aβ, or p‐tau deposits, indicating additional, pathology‐independent effects.

In addition to the *APOE*4 genotype, our combined analysis elevated another initially only suggestive hit to genome‐wide significance. The variant (rs6480922) is located in an intron of the *ZMIZ1* gene that encodes a transcriptional coactivator of various signaling pathways, including, but not limited to, p53, SMAD3, and NOTCH.^29^ While some of these have been described to affect mitochondrial function and/or autophagy,^30^, ^31^ a direct role of ZMIZ1 for mitophagy has not been reported yet. However, intriguingly, inactivation of the zebrafish ortholog leads to reduced expression of autophagy genes and an accumulation of mitochondrial DNA, which could be indicative of mitophagy defects.^32^ A very recent study also just described a positive feedback loop between SMAD3 and PINK1 in the regulation of mitophagy,^33^ providing different mechanisms through which ZMIZ1 may affect p‐S65‐Ub levels.

In humans, pathogenic *ZMIZ1* mutations have been linked to a syndromic neural disorder with intellectual disability and neurodevelopmental delay.^34^ Furthermore, variants in the *ZMIZ1* gene have been identified as risk alleles for various human cancers, autoimmune diseases, and chronic inflammation.^29^ Although we see no evidence of rs6480922 acting as an expression quantitative trait loci status for *ZMIZ1* or neighboring genes, further studies are warranted and should consider cell‐type‐specific effects.^35^ Of note, and in contrast to *APOE*4, *ZMIZ1* rs6480922 was associated with lower p‐S65‐Ub levels, increased brain weight, and reduced neuropathological burden. It is also noteworthy that the *ZMIZ1* rs6480922 effect was stronger in the absence of the *APOE*4 allele. Altogether, the variant may, therefore, reduce p‐S65‐Ub levels directly by improving mitochondrial health or promoting mitophagy flux or indirectly by mitigating neuropathology. Follow‐up studies are required to test the potential functional role(s) of ZMIZ1 during mitophagy or as a resilience factor to mitochondrial damage and LBD neuropathology.

Our study also has certain limitations. First, the analysis only included LBD cases of Caucasian ancestry, and therefore it will be important for future work to assess genetic risk factors for p‐S65‐Ub in other ethnic groups. Second, despite a relatively large sample size for a study of neuropathologically confirmed LBD cases, the possibility of a type II error (i.e., a false‐negative finding) is still important to consider, and emphasis should be placed on 95% confidence limits when interpreting results. We cannot conclude that a true association with p‐S65‐Ub level does not exist simply due to the occurrence of a non‐genome‐wide significant *p* value in our study; it is for this reason that we also highlighted “suggestive” associations that met a less stringent *p* value threshold of *p *< 1 × 10^−6^, and inclusion of more samples will likely reveal additional genome‐wide significant hits. Third, our proof‐of‐concept study focused on p‐S65‐Ub levels in the entire hippocampus. Limiting the analysis to certain vulnerable hippocampal subfields or expansion to different brain regions or even different disease cohorts might shed more light onto shared or distinct aging and neurodegenerative pathways across a spectrum of disorders. Finally, though the focus of our study is on neuropathologically confirmed LBD, it would also have been of interest to examine associations of genetic variants with p‐S65‐Ub levels separately in different clinical diagnosis subgroups (e.g., PD, DLB, AD). However, due to the nature of the study, which included an autopsy cohort of a neuropathologically defined disease obtained from a brain bank, high‐quality information regarding clinical diagnosis that is made consistently according to standard criteria was not available.

Altogether, capitalizing on a specific and sensitive marker for mitochondrial damage and mitophagy alterations, we herein identified *APOE*4 and *ZMIZ1* rs6480922 as new genetic modifiers in a large series of pathologically defined LBD brains. As mitochondrial deficits are early pathological features of LBD, the nominated variants in the current study may ultimately lead to new therapeutic targets that benefit the fast‐growing disease population. With the ongoing development of small‐molecule mitophagy activators (reviewed in Antico et al.^36^), results from this study may help guide future customized therapies for carriers with specific genetic variants. Together with other recent reports,^37^, ^38^ we demonstrated the versatility of the mitophagy tag as a genetic screening tool and further highlighted its relevance and potential as a biomarker of aging and neurodegeneration. Given that p‐S65‐Ub is also present and detectable in human blood,^15^ studies interrogating its suitability to monitor mitochondrial health status in clinical samples are warranted and may help improve diagnosis and prognosis in LBD.

## CONFLICT OF INTEREST STATEMENT

Mayo Clinic, FCF, and WS hold a patent related to PRKN activators (Small Molecule Activators of Parkin Enzyme Function, US Patent 11401255B2; August 2, 2022). All other authors declare they have no competing interests. This research was conducted in compliance with Mayo Clinic conflict of interest policies. Author disclosures are available in the Supporting Information.

## CONSENT STATEMENT

All brain samples were from autopsies performed after approval by the legal next of kin. Consent from human subjects is not required for research on deidentified *post mortem* brain tissue.

## Supporting information



Supporting Information

Supporting Information
