# Supplementary material for: Genome‐wide association analysis identifies APOE as a mitophagy modifier in Lewy body disease
Source: Alzheimers Dement. 2025 May 1;21(4):e70198. doi: 10.1002/alz.70198 (PMC12044520; doi:10.1002/alz.70198)
Supplement: Supplementary file 2 — Supporting Information [file ALZ-21-e70198-s002.pdf]

Genome-wide association analysis identifies APOE as a mitophagy modifier in Lewy body disease

Xu Hou[1,†], Michael G. Heckman[2,†], Fabienne C. Fiesel[1,3], Shunsuke Koga[1], Alexandra I. Soto-Beasley[1], Jens O. Watzlawik[1], Jing Zhao[1], Rebecca R. Valentino[1], Patrick W. Johnson[2], Launia J. White[2], Zachary S. Quicksall[2], Joseph S. Reddy[4], Jose Bras[5,6], Rita Guerreiro[5,6], Na Zhao[1,3], Guojun Bu[1,3], Dennis W. Dickson[1,3], Owen A. Ross[1,3,*], and Wolfdieter Springer[1,3,*]

Author affiliations:

1. Department of Neuroscience, Mayo Clinic, Jacksonville, FL, USA
2. Division of Clinical Trials and Biostatistics, Mayo Clinic, Jacksonville, FL, USA
3. Neuroscience PhD Program, Mayo Clinic Graduate School of Biomedical Sciences, Jacksonville, FL, USA
4. Department of Quantitative Health Sciences, Mayo Clinic, Jacksonville, FL, USA
5. Department of Neurodegenerative Science, Van Andel Institute, Grand Rapids, MI, USA
6. Division of Psychiatry and Behavioral Medicine, Michigan State University College of Human Medicine, Grand Rapids, MI, USA

[†] These authors contributed equally.

* Correspondence should be addressed to:

Wolfdieter Springer PhD
Department of Neuroscience, Mayo Clinic
4500 San Pablo Road, Jacksonville, FL 32224, USA
E-mail: Springer.Wolfdieter@mayo.edu
Tel: +1 904 953 6129
Fax: +1 904 953 7117

Owen A. Ross PhD
Department of Neuroscience, Mayo Clinic
4500 San Pablo Road, Jacksonville, FL 32224, USA
E-mail: Ross.Owen@mayo.edu
Tel: +1 904 953 6280
Fax: +1 904 953 7117

Supplemental Table 1. Subject characteristics

	Discovery series (*n*=754)		Replication series (*n*=258)	
**Variable**	***n***	**Median (minimum, maximum) or No. (%) of cases**	***n***	**Median (minimum, maximum) or No. (%) of cases**
**Age at death (years)**	754	78 (48, 99)	258	76 (56, 95)
**Sex (Male)**	754	450 (59.7%)	258	186 (72.1%)
**LBD subtype**	754		258	
Brainstem		108 (14.3%)		0 (0.0%)
Transitional		263 (34.9%)		91 (35.3%)
Diffuse		383 (50.8%)		167 (64.7%)
**Braak (tau) stage**	754		205	
0		16 (2.1%)		6 (2.9%)
I		32 (4.2%)		10 (4.9%)
II		132 (17.5%)		43 (21.0%)
III		199 (26.4%)		51 (24.9%)
IV		117 (15.5%)		71 (34.6%)
V		109 (14.5%)		14 (6.8%)
VI		149 (19.8%)		10 (4.9%)
**Thal phase**	754		258	
0		101 (13.4%)		42 (16.3%)
1		65 (8.6%)		23 (8.9%)
2		41 (5.4%)		23 (8.9%)
3		151 (20.0%)		68 (26.4%)
4		77 (10.2%)		33 (12.8%)
5		319 (42.3%)		69 (26.7%)
**p-S65-Ub level**	754	3.23 (0.06, 38.02)	258	2.33 (0.11, 25.25)

Supplemental Table 2. Summary of additional neuropathological characteristics

Variable	*n*	Median (minimum, maximum)
Brain weight (g)	1005	1160 (660, 1630)
αSyn burden	717	1.95 (0.18, 63.84)
SP density (count per microscope field)	1004	4.50 (0.00, 38.33)
NFT density (count per microscope field)	1009	2.75 (0.00, 40.50)

Supplemental Table 3a. Genotype counts and frequencies in the discovery series for the seven variants displaying genome-wide significant and suggestive associations

Variant	Minor allele count and frequency	Major allele count and frequency	Genotype 1 count and frequency	Genotype 2 count and frequency	Genotype 3 count and frequency
rs429358	C: 452 (30.0%)	T: 1032 (70.0%)	TT: 373 (49.5%)	TC: 310 (41.1%)	CC: 71 (9.4%)
rs6712544	A: 73 (4.8%)	C: 1435 (95.2%)	CC: 683 (90.6%)	CA: 69 (9.2%)	AA: 2 (0.3%)
rs10935361	T: 463 (30.7%)	C: 1045 (69.3%)	CC: 358 (47.5%)	CT: 329 (43.6%)	TT: 67 (8.9%)
rs157916	G: 723 (47.9%)	A: 785 (52.1%)	AA: 203 (26.9%)	AG: 379 (50.3%)	GG: 172 (22.8%)
rs76354500	C: 57 (3.8%)	T: 1451 (96.2%)	TT: 700 (92.8%)	TC: 51 (6.8%)	CC: 3 (0.4%)
rs6480922	T: 308 (20.4%)	C: 1200 (79.6%)	CC: 474 (62.9%)	CT: 252 (33.4%)	TT: 28 (3.7%)
rs11041236	T: 18 (1.2%)	C: 1490 (98.8%)	CC: 736 (97.6%)	CT: 18 (2.4%)	TT: 0 (0.0%)

Supplemental Table 3b. Genotype counts and frequencies in the replication series for the seven variants selected for inclusion in the replication series

Variant	Minor allele count and frequency	Major allele count and frequency	Genotype 1 count and frequency	Genotype 2 count and frequency	Genotype 3 count and frequency
rs429358	C: 149 (29.1%)	T: 363 (70.9%)	TT: 134 (52.3%)	TC: 95 (37.1%)	CC: 27 (10.5%)
rs6712544	A: 30 (5.8%)	C: 486 (94.2%)	CC: 229 (88.8%)	CA: 28 (10.9%)	AA: 1 (0.4%)
rs10935361	T: 160 (31.0%)	C: 356 (69.0%)	CC: 120 (46.5%)	CT: 116 (45.0%)	TT: 22 (8.5%)
rs157916	G: 243 (47.1%)	A: 273 (52.9%)	AA: 64 (24.8%)	AG: 115 (44.6%)	GG: 79 (30.6%)
rs76354500	C: 21 (4.1%)	T: 491 (95.9%)	TT: 236 (92.2%)	TC: 19 (7.4%)	CC: 1 (0.4%)
rs6480922	T: 106 (20.5%)	C: 410 (79.5%)	CC: 161 (62.4%)	CT: 88 (34.1%)	TT: 9 (3.5%)
rs11041236	T: 10 (1.9%)	C: 506 (98.1%)	CC: 248 (96.1%)	CT: 10 (3.9%)	TT: 0 (0.0%)

Supplemental Table 4. Genome-wide or suggestive associations with p-S65-Ub level - Males

					Discovery series (*n*=450)			Replication series (*n*=186)			Combined analysis (n=636)	
**Chr.**	**Position**	**Variant**	**Gene or closest gene[A]**	**MA**	**MAF**	***β*** **(95% CI)**	**p-value**	**MAF**	***β*** **(95% CI)**	**p-value**	***β*** **(95% CI)**	**p-value**
19	44908684	rs429358	*APOE*	C	27.2%	0.54 (0.39, 0.68)	1.31×10^{-11}	28.3%	0.51 (0.28, 0.74)	1.97×10^{-5}	0.53 (0.41, 0.66)	1.19×10^{-16}
2	116947120	rs6712544	*DPP10 & DDX18*[A]	A	4.4%	-0.68 (-1.03, -0.34)	9.13×10^{-5}	5.6%	-0.25 (-0.76, 0.26)	0.33	-0.51 (-0.92, -0.09)	0.016
3	139926692	rs10935361	*NMNAT3 & CLSTN2*[A]	T	30.4%	-0.23 (-0.38, -0.07)	0.004	30.9%	0.02 (-0.22, 0.27)	0.84	-0.12 (-0.37, 0.12)	0.32
7	130884634	rs157916	*LINC-PINT*	A	47.2%	0.33 (0.19, 0.47)	5.88×10^{-6}	45.2%	-0.03 (-0.25, 0.20)	0.81	0.16 (-0.19, 0.51)	0.37
8	14866463	rs76354500	*SGCZ*	C	3.4%	-0.70 (-1.08, -0.33)	2.17×10^{-4}	3.5%	-0.34 (-0.96, 0.28)	0.28	-0.60 (-0.93, -0.28)	0.0003
10	79154025	rs6480922	*ZMIZ1*	T	20.4%	-0.42 (-0.59, -0.24)	5.08×10^{-6}	20.4%	-0.22 (-0.50, 0.07)	0.13	-0.35 (-0.54, -0.17)	0.0002
11	7194782	rs11041236	*RBMXL2 & MIR302E*[A]	T	1.4%	-1.17 (-1.76, -0.58)	1.29×10^{-4}	2.4%	0.52 (-0.22, 1.26)	0.17	-0.34 (-2.00, 1.32)	0.69

Chr=Chromosome. MA=minor allele; MAF=minor allele frequency; β=regression coefficient; CI=confidence interval. For the discovery series and the replication series, β values, 95% CIs, and p-values result from linear regression models that were adjusted for age at death, hippocampal region, and the top five principal components of genetic data (the top five principal components were adjusted for only in the discovery series). β values are interpreted as the change in mean p-S65-Ub level (on the natural logarithm scale) corresponding to each additional minor allele for the given variant. For the combined analysis, β values, 95% CIs, and p-values result from a random effects meta-analysis. [A]The closest genes to the identified variant.

Supplemental Table 5. Genome-wide or suggestive associations with p-S65-Ub level - Females

					Discovery series (*n*=304)			Replication series (*n*=72)			Combined analysis (n=376)	
**Chr.**	**Position**	**Variant**	**Gene or closest gene[A]**	**MA**	**MAF**	***β*** **(95% CI)**	**p-value**	**MAF**	***β*** **(95% CI)**	**p-value**	***β*** **(95% CI)**	**p-value**
19	44908684	rs429358	*APOE*	C	34.1%	0.46 (0.29, 0.64)	2.65×10^{-7}	31.3%	0.52 (0.09, 0.95)	0.019	0.47 (0.31, 0.63)	6.49×10^{-9}
2	116947120	rs6712544	*DPP10 & DDX18*[A]	A	5.4%	-0.62 (-0.99, -0.25)	0.001	6.3%	0.42 (-0.42, 1.27)	0.32	-0.17 (-1.18, 0.84)	0.74
3	139926692	rs10935361	*NMNAT3 & CLSTN2*[A]	T	31.1%	-0.47 (-0.65, -0.29)	7.27×10^{-7}	31.3%	-0.22 (-0.67, 0.22)	0.32	-0.43 (-0.61, -0.26)	8.90×10^{-7}
7	130884634	rs157916	*LINC-PINT*	A	49.0%	0.20 (0.03, 0.37)	0.020	52.1%	-0.11 (-0.48, 0.26)	0.56	0.09 (-0.20, 0.38)	0.54
8	14866463	rs76354500	*SGCZ*	C	4.3%	-0.74 (-1.15, -0.34)	3.62×10^{-4}	5.6%	-0.25 (-1.04, 0.54)	0.53	-0.62 (-1.04, -0.20)	0.0038
10	79154025	rs6480922	*ZMIZ1*	T	20.4%	-0.28 (-0.49, -0.07)	0.010	20.8%	-0.26 (-0.78, 0.25)	0.31	-0.28 (-0.47, -0.08)	0.0053
11	7194782	rs11041236	*RBMXL2 & MIR302E*[A]	T	0.8%	-1.60 (-2.53, -0.67)	8.85×10^{-4}	0.7%	1.22 (-1.26, 3.69)	0.33	-0.43 (-3.16, 2.29)	0.75

Chr=Chromosome. MA=minor allele; MAF=minor allele frequency; β=regression coefficient; CI=confidence interval. For the discovery series and the replication series, β values, 95% CIs, and p-values result from linear regression models that were adjusted for age at death, hippocampal region, and the top five principal components of genetic data (the top five principal components were adjusted for only in the discovery series). β values are interpreted as the change in mean p-S65-Ub level (on the natural logarithm scale) corresponding to each additional minor allele for the given variant. For the combined analysis, β values, 95% CIs, and p-values result from a random effects meta-analysis. [A]The closest genes to the identified variant.

Supplemental Table 6. Association between *APOE* genotype (as defined by rs429358 and rs7412) and p-S65-Ub level

APOE genotype	*n*	Median (minimum, maximum) p-S65-Ub level	*β* (95% CI)	p-value
**Discovery series**				
ε2/ε3	44	1.08 (0.12, 23.16)	-0.36 (-0.68, -0.04)	0.028
ε2/ε4	23	1.03 (0.16, 17.07)	-0.47 (-0.90, -0.04)	0.031
ε3/ε3	329	2.14 (0.06, 27.19)	0.00 (reference)	N/A
ε3/ε4	287	4.61 (0.14, 38.02)	0.55 (0.39, 0.71)	3.64×10^{-11}
ε4/ε4	71	5.81 (0.74, 19.19)	0.95 (0.69, 1.21)	3.43×10^{-12}
**Replication series**				
ε2/ε3	16	1.59 (0.61, 8.54)	0.02 (-0.55, 0.59)	0.94
ε2/ε4	12	1.00 (0.40, 9.79)	-0.18 (-0.83, 0.48)	0.60
ε3/ε3	118	1.54 (0.20, 20.69)	0.00 (reference)	N/A
ε3/ε4	83	3.25 (0.11, 25.25)	0.48 (0.17, 0.80)	0.0027
ε4/ε4	27	5.98 (0.96, 24.36)	1.15 (0.69, 1.61)	1.48×10^{-6}
**Combined analysis**				
ε2/ε3	60	1.33 (0.12, 23.16)	-0.25 (-0.59, 0.09)	0.16
ε2/ε4	35	1.03 (0.16, 17.07)	-0.38 (-0.74, -0.02)	0.037
ε3/ε3	447	2.01 (0.06, 27.19)	0.00 (reference)	N/A
ε3/ε4	370	4.35 (0.11, 38.02)	0.54 (0.39, 0.68)	1.59×10^{-13}
ε4/ε4	98	5.88 (0.74, 24.36)	1.00 (0.77, 1.22)	5.35×10^{-18}

β=regression coefficient; CI=confidence interval. For the discovery series and the replication series, β values, 95% CIs, and p-values result from linear regression models that were adjusted for age at death, sex, hippocampal region, and the top five principal components of genetic data (the top five principal components were adjusted for only in the discovery series). β values are interpreted as the difference in mean p-S65-Ub level (on the natural logarithm scale) in comparison to the *APOE* ε3/ε3 reference group. For the combined analysis, β values, 95% CIs, and p-values result from a random effects meta-analysis.

Supplemental Table 7. Associations of *APOE* rs429358 and *ZMIZ1* rs6480922 with additional neuropathological characteristics

		Association with *APOE* rs429358		Association with *ZMIZ1* rs6480922	
**Variable**	***n***	***β* (95% CI)**	**p-value**	***β* (95% CI)**	**p-value**
Brain weight (g)	1005	-32.57 (-45.34, -19.81)	6.47×10^{-7}	26.19 (11.35, 41.03)	0.0006
αSyn intensity	717	0.20 (0.11, 0.30)	3.59×10^{-5}	-0.12 (-0.22, -0.01)	0.031
SP density	1004	0.54 (0.47, 0.62)	2.31×10^{-41}	-0.21 (-0.30, -0.12)	1.41×10^{-5}
NFT density	1009	0.33 (0.27, 0.39)	7.36×10^{-23}	-0.13 (-0.21, -0.05)	0.001

β=regression coefficient; CI=confidence interval. β values, 95% CIs, and p-values result from linear regression models that were adjusted for age at death and sex. β values are interpreted as the change in the mean neuropathological variable (on the natural logarithm scale for αSyn burden and on the cube root scale for SP and NFT density) corresponding to each additional minor allele for the given variant.

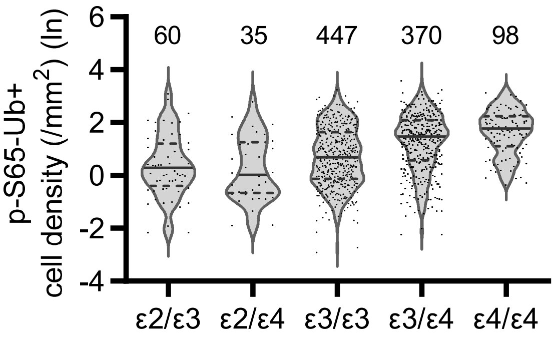

Supplemental Figure 1. The effect of *APOE* genotypes on p-S65-Ub levels in LBD autopsy brain. Violin plot of pS65-Ub level (natural logarithm scale) according to *APOE* genotypes (i.e., ε2/ε3, ε2/ε4, ε3/ε3, ε3/ε4, ε4/ε4; there were no ε2/ε2 genotypes in our study) in the combined (discovery and replication) series. Sample size is indicated on top of each genotype group.
